# The Volatilomic Footprints of Human HGC-27 and CLS-145 Gastric Cancer Cell Lines

**DOI:** 10.3389/fmolb.2020.607904

**Published:** 2021-01-11

**Authors:** Andreas Leiherer, Daria Ślefarska, Marcis Leja, Christine Heinzle, Axel Mündlein, Ilze Kikuste, Linda Mezmale, Heinz Drexel, Chris A. Mayhew, Paweł Mochalski

**Affiliations:** ^1^Vorarlberg Institute for Vascular Investigation and Treatment (VIVIT), Feldkirch, Austria; ^2^Private University of the Principality of Liechtenstein, Triesen, Liechtenstein; ^3^Medical Central Laboratories, Feldkirch, Austria; ^4^Institute for Breath Research, University of Innsbruck, Dornbirn, Austria; ^5^Institute of Chemistry, Jan Kochanowski University, Kielce, Poland; ^6^Institute of Clinical and Preventive Medicine, University of Latvia, Riga, Latvia; ^7^Faculty of Medicine, University of Latvia, Riga, Latvia; ^8^Riga East University Hospital, Riga, Latvia; ^9^Drexel University College of Medicine, Philadelphia, PA, United States; ^10^Molecular Physics Group, School of Physics and Astronomy, University of Birmingham, Birmingham, United Kingdom

**Keywords:** volatile organic compounds, GC-MS, chemical footprint, gastric cancer, CLS-145, HGC-27

## Abstract

The presence of certain volatile biomarkers in the breath of patients with gastric cancer has been reported by several studies; however, the origin of these compounds remains controversial. *In vitro* studies, involving gastric cancer cells may address this problem and aid in revealing the biochemical pathways underlying the production and metabolism of gastric cancer volatile indicators. Gas chromatography with mass spectrometric detection, coupled with headspace needle trap extraction as the pre-concentration technique, has been applied to map the volatilomic footprints of human HGC-27 and CLS-145 gastric cancer cell lines and normal Human Stomach Epithelial Cells (HSEC). In total, 27 volatile compounds are found to be associated with metabolism occurring in HGC-27, CLS-145, and HSEC. Amongst these, the headspace concentrations of 12 volatiles were found to be reduced compared to those above just the cultivating medium, namely there was an observed uptake of eight aldehydes (2-methylpropanal, 2-methyl-2-propenal, 2-methylbutanal, 3-methylbutanal, hexanal, heptanal, nonanal, and benzaldehyde), three heterocyclic compounds (2-methyl-furan, 2-ethyl-furan, and 2-pentyl-furan), and one sulfur-containing compound (dimethyl disulphide). For the other 15 volatiles, the headspace concentrations above the healthy and cancerous cells were found to be higher than those found above the cultivating medium, namely the cells were found to release three esters (ethyl acetate, ethyl propanoate, and ethyl 2-methylbutyrate), seven ketones (2-pentanone, 2-heptanone, 2-nonanone, 2-undecanone, 2-tridecanone, 2-pentadecanone, and 2-heptadecanone), three alcohols (2-methyl-1-butanol, 3-methyl-1-butanol, and 2-ethyl-1-hexanol), one aromatic compound (toluene), and one sulfur containing compound [2-methyl-5-(methylthio) furan]. In comparison to HSEC, HGC-27 cancer cell lines were found to have significantly altered metabolism, manifested by an increased production of methyl ketones containing an odd number of carbons. Amongst these species, three volatiles were found exclusively to be produced by this cell line, namely 2-undecanone, 2-tridecanone, and 2-heptadecanone. Another interesting feature of the HGC-27 footprint is the lowered level of alcohols and esters. The CLS-145 cells exhibited less pronounced changes in their volatilomic pattern compared to HSEC. Their footprint was characterized by the upregulated production of esters and 2-ethyl-hexanol and downregulated production of other alcohols. We have therefore demonstrated that it is possible to differentiate between cancerous and healthy gastric cells using biochemical volatile signatures.

## Introduction

There is growing interest in the human volatilome as a powerful tool capable of providing novel biomarkers for medical diagnosis and therapy monitoring (Beauchamp et al., 2020). The volatilome is defined as a subset of the metabolome embracing volatile organic compounds (VOCs) of various origins within the human organism. Human VOCs can have a systemic origin or stem from exogenous sources such as environmental exposure, diet, or microbiota activity (Amann et al., 2014), resulting in specific biochemical signatures, which can be altered by abnormal processes occurring in the organism, including metabolic disorders, cancer, or other diseases. These alterations can stem from changes in enzyme activity, modifications of proteins, or activation of genes, for examples. The analysis of biochemical volatile footprints in breath, skin emanations, and in other bodily fluids, such as urine, saliva, or sweat, provides a unique opportunity to monitor microbiota activity, individuals' exposure to environmental toxins, or for use in screening for various diseases, including cancer (Haick et al., 2014; Broza et al., 2015; del Rio et al., 2015; Beauchamp et al., 2020). This volatilomic approach suffers, however, from a number of limitations, which constrict its application within a clinical setting. The main unresolved issue here is the often poor biochemical understanding of the origin, behavior and metabolic fate of volatile biomarkers in the human organism.

*In vitro* studies, involving cell cultures and microorganisms, are of considerable use in revealing the biochemical pathways underlying the production and metabolism of volatile markers and, thereby, can help address the aforementioned problems. Indeed, over the last decade a substantial effort has been made to map chemical signatures of human cell lines. A particular focus has been on cancers, including lung (Filipiak et al., 2008, 2010; Sponring et al., 2009, 2010; Wang et al., 2012; Schallschmidt et al., 2015), liver (Mochalski et al., 2013b), breast (Silva et al., 2017), skin (Kwak et al., 2013), colon (Zimmermann et al., 2007), and bladder (Rodrigues et al., 2018).

Gastric cancer is the second most frequent cause of cancer-associated death worldwide, being highly aggressive and promoting distant metastasis, with typical metastatic sites being the lungs, liver and bones (Jmour et al., 2017).

A number of studies aimed at the identification of volatile markers of gastric cancer in different bodily fluids and tissues have bene published. Kumar et al. (2012) investigated the head-space of gastric juice using Selected Ion Flow Tube Mass Spectrometry (SIFT-MS) and identified seven volatiles namely: acetone, formaldehyde, acetaldehyde, hexanoic acid, hydrogen sulfide, hydrogen cyanide, and methyl phenol, which showed differences in their headspace levels between cancer (19 patients) and healthy (11 patients) subjects. In a follow-up study, Kumar et al. (2015) investigated the value of breath volatiles to identify esophageal and gastric adenocarcinoma. In that study, they reported 12 compounds (pentanoic acid, hexanoic acid, phenol, methyl phenol, ethyl phenol, butanal, pentanal, hexanal, heptanal, octanal, nonanal, and decanal) showing significantly higher concentrations (*p* < 0.05) in the gastric cancer patients than in the noncancer controls. Durán-Acevedo et al. (2018) employed in parallel gold nanoparticles (AuNP) gas sensor and gas chromatography mass spectrometry (GC-MS) to determine gastric cancer signatures in human breath. The GC-MS study resulted in the recognition of six VOCs that showed statistically significant differences between the cancer patients (*n* = 14) and the control group (*n* = 15). Amongst these species, the concentration of four (octadecane, m-xylene, hexadecane, trans-2,2-dimethyl-3-decene) were found to be increased in the breath of gastric cancer patients, and the concentration of two [eicosane and 1-cyclohexyl-2-(cyclohexylmethyl) pentane] decreased. A classification model based on principal component analysis (PCA) and employing GC-MS abundancies of these volatiles provided 90% accuracy, 93% sensitivity, and 87% specificity. Moreover, the PCA analysis employing six features of the gas sensor signal resulted in a classification yielding 97% accuracy, 100% sensitivity, and 93% specificity. Xu et al. (2013), using GC-MS, found that five volatile markers (2-butoxy-ethanol, 2-propenenitrile, 6-methyl-5-hepten-2-one, isoprene, and furfural) were increased in the exhaled air of subjects with gastric cancer and/or peptic ulcer, as compared with less severe gastric conditions. In the same study, they showed that breath footprints detected by nanomaterial-based sensors could be used for identification of gastric cancer and distinction from benign stomach ulcers and less severe stomach conditions. In a further study (Amal et al., 2015), eight breath species (furfural, 2-propenenitrile, hexadecane, 2-butoxy-ethanol, 1,2,3-tri-methylbenzene, 4-methyloctane, 2-butanone, and α-methyl-styrene), identified using GC-MS, were found to differ significantly between gastric cancer and control groups. More recently, Nakhleh et al. (2017) showed that an artificially intelligent nanoarray, based on molecularly modified gold nanoparticles, can detect and discriminate between 17 different disease conditions, including gastric cancer from exhaled breath. Buszewski et al. (2008) investigated the VOCs released by gastric cancer and normal tissues obtained from five patients. The levels of carbon disulfide and 1-propanol were found to be increased in the headspace of cancer tissue as compared to the healthy one. In a recent study (Mochalski et al., 2018), we compared the volatilomic footprint of cancer and normal tissues collected from 41 patients during gastric surgery. The emission of four species (carbon disulfide, pyridine, 3-methyl-2-butanone, and 2-pentanone) was found to be significantly higher from cancer tissue than from healthy tissue. When it comes to cell lines, the volatilomic footprints of gastric cancer cells MGC-803 and GES-1 gastric mucous cells were investigated by Zhang et al. (2014). Eight volatile species were associated with the metabolism of the lines under study. Two volatiles 3-octanone and 2-butanone were found exclusively to be produced by the MGC-803 cells; whereas, a further three species (formic acid propyl ester, 1.4-butanediol, and 2, 6, 11-trimethyl dodecane) were detected solely in the headspace of the GES-1 cells. The remaining three volatiles (4-isopropoxybutanol, nonanol, and 4-butoxy-1-butanol) were released by both lines; however, their emission was significantly lower in cancer cells in comparison to the normal ones.

Within this study, we focus on the volatilomic footprints of human HGC-27 and CLS-145 gastric cancer cell lines and normal Human Stomach Epithelial Cells (HSEC). In particular, this involves determining volatiles that are produced (release) and used (uptake) through metabolic processes of the cells, and identifying changes in VOCs production that are caused by the cancer. This new knowledge will aid in the discovery of volatile biomarkers in breath or contained in the headspace of urine, and hence support studies that are aiming to provide the volatile signatures for use in noninvasively detecting people with gastric cancer. To the best of our knowledge, the VOC profiles of the HGC-27 and CLS-145 are being reported for the first time. For this study, headspace needle trap extraction (HS-NTE), as the pre-concentration method, and GC-MS have been applied to capture and analyze, respectively, the headspace above the cultivating medium and cells in the cultivating medium.

## Materials and Methods

### Chemicals and Standards

Calibration mixtures were produced from liquid substances using a protocol outlined in detail elsewhere (Mochalski et al., 2012, 2013a). Therefore, only a short description of the procedure is provided here. The reference substances, with purities ranging from 95 to 99.9%, were purchased from Sigma-Aldrich (Austria) and Fluka (Switzerland). Gaseous calibration mixtures were prepared by injecting and evaporating a few microliters of liquid or gaseous analyte into evacuated glass bulbs (Supelco, Canada). The desired calibration levels were achieved by transferring appropriate volumes of the bulb standard into Tedlar bags (SKC Inc., USA) filled with predefined amounts of humidified zero air (RH 100% at 37°C). Effectively, gas mixtures with VOCs volume fractions ranging from 10 ppt to 160 ppb were used for calibration. Calibration curves were obtained on the basis of 6–7 distinct and independent concentration levels.

### Cell Cultivation

The CLS-145 cell line was derived from fragments of a gastric papillary adenocarcinoma of pars cardiac resected from a female therapy-naïve patient. The HGC 27 cell line was established from a gastric cancer patient with a histologically diagnosed undifferentiated carcinoma as described elsewhere (Akagi and Kimoto, 1976). Human stomach epithelial cells (HSEC; CSC-C9230J) are noncarcinoma cells, isolated from normal human stomach tissue, which served as the controls. Gastric cancer cell lines CLS-145 and HGC 27 were purchased from CLS cell lines service GmbH (Eppelheim, Germany). Noncancer cells HSEC were purchased from Creative Bioarray (Shirley, USA). All cells were checked for mycoplasmic contaminations using MycoSPY®-PCR-Kit (Biontex, Munich, Germany). Cells were cultivated at 37°C and 5% CO_2_ in medium containing 50% SuperCult®Complete Human Epithelial Cell Medium (Creative Bioarray), 44.5% DMEM/Ham's F12 (1:1) medium (Thermo Fisher Scientific, Waltham, USA), 5% FCS (Thermo Fisher Scientific), and 0.5% Penicillin/Streptomycin (Thermo Fisher Scientific). Doubling time for the cells using the above cultivating condition was about 30, 46, and 100 h for HGC-27, HSEC, and CLS-145, respectively. Cultivation was done in glass flasks (Ruprechter, Austria) occupying a volume of 21 × 5.5 × 11.5 cm^3^ (1 L nominal volume, bottom area of ~240 cm^2^). They were grown exponentially and thus were asynchronous with respect to the cell cycle stage. A more detailed description of cultivation is given elsewhere (Mochalski et al., 2013b, 2014). For each experiment, all three-cell cultures, as well as the medium control, were analyzed in triplicates (three flasks). One sample of the headspace gas per flask was taken. Four independent experiments were performed at different time points and handled by three different researchers. Apart from that, all cultures and controls were prepared and cultivated under identical conditions using the same protocols, materials, and reagents.

### HS-NTE Sampling Protocol

A detailed description of the extraction protocol has been given elsewhere (Mochalski et al., 2013a), and therefore only a brief outline of the procedure and its modifications will be given here. VOCs were extracted from the headspace (HS) of the cultivating medium and above cell cultures in the cultivating medium, using needle trap extraction (HS-NTE). Two-bed 23-gauge Silcosteel-treated stainless steel needle trap devices (NTDs) (2 cm of Carbopack X and 1 cm of Carboxen 1000, both 60/80 mesh, PAS Technology, Germany) were employed for this purpose. Prior to their use, all NTDs were pre-conditioned at 290°C under the flow of high-purity nitrogen (99.9999%) for 10 mins. The NTE was performed by inserting a NTD via a septum into a cultivation flask and drawing 100 mL of the headspace gas at a constant flow rate of 3 mL/min. After extraction, the needles were introduced into the inlet of the gas chromatograph, where the trapped volatiles were thermally desorbed at 290°C in a splitless mode. In parallel, one blank sample containing nitrogen was analyzed using the same protocol to identify possible contaminants stemming from sources other than cells/medium. The resulting concentration levels were subtracted (if applicable) from the respective values in the associated headspace samples.

### GC-MS Analysis

The headspace samples were analyzed using an Agilent 7890A/5975C GC-MS instrument (Agilent, USA). The GC inlet, equipped with an inert SPME liner (0.75 mm inner diameter, Supelco, Canada), worked in the splitless mode (1 min), followed by a split mode (ratio of 1:50). VOCs were separated using an Rxi-624Sil MS column (30 m × 0.32 mm, film thickness 1.8 μm, Restek, USA) operating in a constant flow of helium at 1.5 mL × min^−1^. The oven temperature program was set at 40°C for 10 min, 5°C min^−1^ to 150, 150°C for 5 mins, 10°C × min^−1^ to 280°C, and 280°C for 10 min. The mass spectrometer worked in a synchronous SCAN/SIM mode. The SCAN mode, with *m/z* ranging from 20 to 250, was used for the untargeted analysis of VOCs and the quantification of more abundant species. In the case of the latter, the peak integration was based on extracted *m/z* ratio chromatograms. The proper selection of *m/z* values allowed for a separation of the majority of peaks of interest from their neighbors. Selected VOCs were quantified using the SIM (selective ion monitoring) mode. The applied SCAN quantifier ions and SIM *m/z* values, and dwell times are given in Table 1. The quadrupole, ion source, and transfer line temperatures were kept at 150, 230, and 280°C, respectively.

**Table 1:** Retention times (*R*_*t*_) [min], quantifier ions, SIM dwell times [μs], LODs [ppb], RSDs (%), coefficients of variation (*R*^2^), and linear ranges [ppb] for compounds of interest.

**VOC**	**CAS**	***R_***t***_* [min]**	**Quantifier ion (SIM dwell time [μs])**	**LOD [ppb]**	**RSD [%]**	***R*^**2**^**	**Linear range [ppb]**
Propanal, 2-methyl	78-84-2	4.12	72	0.07	4.5	0.997	0.21–38
2-Propenal, 2-methyl-	78-85-3	4.4	70	0.08	8.7	0.995	0.25–22
2-Methylfuran	534-22-5	4.83	82 (80)	0.02	3.7	0.995	0.06–10
Ethyl acetate	141-78-6	5.7	43	0.03	9.0	0.992	0.08–9.5
Butanal, 3-methyl-	590-86-3	8.43	41	0.06	4.6	0.998	0.17–160
Butanal, 2-methyl-	96-17-3	8.98	57	0.08	3.4	0.998	0.24–46
Furan, 2-ethyl-	3208-16-0	10.11	81	0.01	4.6	0.973	0.02–8.5
2-Pentanone	107-87-9	11.08	43	0.01	4.6	0.996	0.03–8.6
Ethyl propanoate	105-37-3	11.8	57 (80)	0.06	4.9	0.993	0.18–8.0
Dimethyl disulphide (DMDS)	624-92-0	13.8	94	0.02	5.2	0.974	0.05–10.2
Toluene	108-88-3	15	91	0.27	4.0	0.991	0.8–30
1-Butanol, 3-methyl-	123-51-3	15.24	70 (80)	0.16	6.0	0.993	0.46–32
1-Butanol, 2-methyl-	137-32-6	15.41	70 (80)	0.15	6.5	0.988	0.45–20
Hexanal	66-25-1	17.75	56	0.15	5.5	0.994	0.45–4
Ethyl 2-methylbutyrate	7452-79-1	19.6	102 (80)	0.01	4.6	0.957	0.03–16
2-Heptanone	105-42-0	22.18	58 (80)	0.08	3.9	0.988	0.25–12
Heptanal	111-71-7	22.49	70	0.08	12	0.987	0.22–3.5
*2-Methyl-5-(methyl thio) furan*	*2371*-*70*-*2*	*23.94*	*128*	–	–	–	–
2-Pentylfuran	3777-69-3	25.18	81	0.03	6.4	0.999	0.07–11
Benzaldehyde	100-52-7	25.5	105	0.03	9.1	0.991	0.09–3
1-Hexanol, 2-ethyl-	104-76-7	27.66	57	0.18	3.7	0.992	0.52–81
2-Nonanone	821-55-6	29.67	58 (80)	0.17	9.0	0.986	0.5–29
Nonanal	124-19-6	29.98	57	0.1	16	0.996	0.3–12
2-Undecanone	112-12-9	37.07	58 (80)	0.03	6.6	0.990	0.09–16
2-Tridecanone	593-08-8	42.83	58 (80)	0.3	15	0.989	1–35
*2-Pentadecanone*	*2345-28-0*	*46.05*	*58 (80)*	–	–	–	–
*2-Heptadecanone*	*2922-51-2*	*48.53*	*58 (80)*	–	–	–	–

*The compounds in italics were not quantified for reasons mentioned in the text; however, the retention times of 2-pentadecanone and 2-heptadecanone were confirmed using the reference standards. Compounds are ordered with respect to increasing retention time*.

A two-step VOCs identification protocol was applied. First, the spectrum of an unknown peak was checked against the NIST mass spectral library. Next, the NIST identification was confirmed by comparing the retention times of peaks of interest with retention times obtained for reference standards prepared as outlined above. All measurement data were generated in four independent experiments, taking place at four different time points in 2020, and were performed by two different analysts.

The production and uptake of volatiles by the HGC-27, CLS-145, and HSEC cells were evaluated using a Wilcoxon signed rank test, and a *p* < 0.05 was taken as being significant. For the purpose of the Wilcoxon test, the left-censored data were estimated by a value LOQ/√2, or LOD/√2 (Antweiler, 2015).

## Results

### Validation Parameters

The validation parameters of the NTE-HS-GC-MS are provided in Table 1. Limits of detection (LODs) were calculated using the algorithm proposed by Huber (2003), and the standard deviation of five consecutive blank signals. The limit of quantification (LOQ) is defined as 3 × LOD. The LOD ranges from 0.01 to 0.27 ppb. Relative standard deviations (RSDs) varied from 3.4 to 16%, which are considered adequate for the purposes of this study. The instrument response was found to be linear within the investigated concentration ranges, with coefficients of determination ranging from 0.957 to 0.999.

### Cell Cultures

Cell cultures were all tested to be free of mycoplasma contamination. They were grown exponentially, and as such were asynchronous. The total number of cells in the measurement flasks at the time of measurement is given in Table 2. Live-dead staining revealed >99% living cells. Thus, the applied experimental procedure did not affect the cells' viability.

**Table 2:** Total number of cells [ ×10^6^] in the cultivation flasks at the time of the measurement.

**Cell line**	**Experiment/flask**											
	**I**			**II**			**III**			**IV**		
	**A**	**B**	**C**	**A**	**B**	**C**	**A**	**B**	**C**	**A**	**B**	**C**
HSEC	10.3	8.3	8.1	36.1	24.1	30.0	16.7	27.0	24.4	27.4	26.4	24.7
CLS-145	2.7	3.2	2.9	–	20.0	15.6	10.1	6.1	9.2	20.0	20.4	21.1
HGC-27	9.8	9.5	9.0	14.7	20.7	31	50.5	44.8	46.3	32.6	30.1	32.1

*Cell numbers are given as triplicates (A–C) of four independent experiments (I–IV). Replicate CLS-145 IIA failed due to some technical problems*.

### Volatilomic Patterns of HGC-27, CLS-145, and HSEC

Amongst the volatiles detected, 27 showed consistent differences in their headspace concentrations compared to those above the cultivation medium only. Of these, 12 compounds were found to have reduced headspace concentrations, and hence the other showed increases in headspace concentrations. Their detection and quantification incidences, as well as their concentrations in the headspace of the cultivation flasks are shown in Table 3; whereas the output of the Wilcoxon signed rank test is presented in Table 4. Those VOCs with increased concentrations (release) are three esters (ethyl acetate, ethyl propanoate, and ethyl 2-methylbutyrate), seven ketones (2-pentanone, 2-heptanone, 2-nonanone, 2-undecanone, 2-tridecanone, 2-pentadecanone, and 2-heptadecanone), three alcohols (2-methyl-1-butanol, 3-methyl-1-butanol, and 2-ethyl-1-hexanol), one aromatic compounds compound (toluene) and one sulfur containing compound [2-methyl-5-(methylthio) furan]. The metabolized species (uptake) comprise of eight aldehydes (2 methylpropanal, 2-methyl-2-propenal, 2-methylbutanal, 3-methylbutanal, hexanal, heptanal, nonanal, and benzaldehyde), three heterocyclic compounds (2-methyl-furan, 2-ethyl-furan, and 2-pentyl-furan) and one sulfur containing compound (dimethyl disulphide). An effort was made to quantify the levels of the aforementioned species in the headspace. However, three compounds [2-methyl-5-(methylthio)-furan, 2-pentadecanone, and 2-heptadecanone] were not quantified owing either to the unavailability of pure substances or problems related to the preparation of reliable reference mixtures. In the case of these species, their levels were assessed only on the basis of peak areas.

**Table 3:** Detection (n_d_) and quantification (n_q_) incidences, concentration ranges, and medians of VOCs in the headspace of only media and cell cultures plus media.

	**VOC**	**CAS**	**HGC−27**		**CLS−145**		**HSEC**		**Medium**	
			**Incidence n_**d**_(n_**q**_)**	**Range (median) [ppb]**	**Incidence n_**d**_(n_**q**_)**	**Range (median) [ppb]**	**Incidence n_**d**_(n_**q**_)**	**Range (median) [ppb]**	**Incidence n_**d**_(n_**q**_)**	**Range (median) [ppb]**
Uptake	Propanal, 2-methyl	78-84-2	12(12)	0.23–0.64 (0.43)	10(7)	0.22–0.72 (0.34)	12(12)	0.38–2.22 (0.66)	12(12)	2.72–44 (10.9)
	2-Propenal, 2-methyl-	78-85-3	6(1)	0.34–0.34 (0.34)	5(1)	0.32–0.32 (0.32)	6(4)	0.46–3.18 (0.88)	12(12)	1.1–20 (1.6)
	2-Methylfuran	534-22-5	12(12)	0.14–0.77 (0.45)	11(8)	0.09–0.37 (0.21)	12(10)	0.09–1.08 (0.45)	12(12)	0.08–1.7 (0.36)
	Butanal, 3-methyl-	590-86-3	7(7)	0.19–1.09 (0.25)	9(6)	0.19–0.96 (0.43)	12(12)	0.26–1.85 (0.53)	12(12)	6.3–153 (50)
	Butanal, 2-methyl-	96-17-3	12(12)	0.29–1.74 (0.47)	11(7)	0.26–0.90 (0.42)	12(10)	0.24–1.10 (0.82)	12(12)	2.42–21 (11)
	Furan, 2-ethyl-	3208-16-0	12(12)	0.06–0.17 (0.12)	11(11)	0.02–0.23 (0.06)	12(12)	0.02–0.27 (0.08)	12(12)	0.11–1.13 (0.3)
	Dimethyl disulphide (DMDS)	624-92-0	2(0)	–	3(3)	0.17–0.29 (0.24)	6(4)	0.06–0.41 (0.38)	8(5)	1.1–62 (1.18)
	Hexanal	66-25-1	10(3)	0.61–0.75 (0.63)	8(2)	0.47–0.57 (0.52)	8(3)	0.72–0.94 (0.74)	12(11)	0.47–4.42 (0.89)
	Heptanal	111-71-7	12(12)	0.27–0.76 (0.51)	11(9)	0.28–0.58 (0.49)	12(10)	0.27–0.92 (0.42)	12(11)	0.48–2.82 (1.1)
	2–Pentylfuran	3777-69-3	12(12)	0.11–0.64 (0.34)	11(11)	0.11–1.43 (0.21)	12(9)	0.10–1.29 (0.46)	12(12)	1.14–1.82 (0.44)
	Benzaldehyde	100-52-7	12(12)	0.09–1.70 (0.21)	11(8)	0.10–1.58 (0.19)	12(8)	0.10–1.74 (0.25)	12(12)	0.12–3.1 (0.6)
	Nonanal	124-19-6	12(10)	0.38–1.16 (0.62)	9(8)	0.45–1.17 (0.55)	12(6)	0.71–1.15 (0.83)	12(9)	0.48–2.23 (1.3)
Release	Ethyl acetate	141-78-6	12(12)	0.25–5.03 (1.48)	11(11)	0.35–4.80 (1.69)	12(12)	0.50–5.49 (2.19)	12(11)	0.09–1.3 (0.32)
	2-Pentanone	107-87-9	12(12)	0.23–1.31 (0.74)	11(11)	0.12–1.28 (0.33)	12(12)	0.24–1.17 (0.45)	12(12)	0.09–0.82 (0.18)
	Ethyl propanoate	105-37-3	12(12)	0.43–2.08 (0.93)	11(11)	0.52–2.84 (1.44)	12(12)	0.92–4.45 (1.71)	9(6)	0.28–2.35 (1.53)
	Toluene	108-88-3	12(12)	2.52–24.21 (6.01)	11(9)	1.00–10.81 (5.07)	12(10)	0.83–25.83 (4.26)	12(11)	1.03–16.4 (4.9)
	1-Butanol, 3-methyl-	123-51-3	12(11)	0.53–1.15 (0.85)	11(11)	0.53–1.54 (0.82)	12(12)	3.62–49.53 (8.29)	8(4)	0.48–0.7 (0.63)
	1-Butanol, 2-methyl-	137-32-6	12(10)	0.55–1.56 (0.98)	10(9)	0.51–1.24 (0.65)	12(12)	1.37–21.11 (5.47)	11(6)	0.75–1.16 (0.76)
	Ethyl 2-methylbutyrate	7452-79-1	12(12)	1.07–15.17 (4.03)	11(11)	0.70–3.66 (1.65)	12(12)	0.68–4.95 (2.20)	5(3)	0.03–0.06 (0.04)
	2-Heptanone	105-42-0	12(12)	1.25–13.05 (2.48)	11(11)	0.25–1.30 (0.48)	12(6)	0.62–2.47 (1.32)	10(7)	0.27–0.57 (0.43)
	*2*-*Methyl*-*5*- *(methyl thio) furan*	*2371-70-2*	*12(12)*	*22,200–107,900 (47,000)*	*11(11)*	*1,300–16,250 (5,100)*	12(12)	4,300–57,500 (22,900)	8(8)	30–630 (100)
	1-Hexanol, 2-ethyl-	104-76-7	12(12)	1.78–21.04 (3.43)	11(11)	2.06–62.64 (6.25)	12(12)	2.32–22.77 (5.30)	12(11)	0.63–21.5 (2.77)
	2-Nonanone	821-55-6	12(12)	3.48–36.03 (8.79)	11(3)	0.92–1.27 (1.02)	6(5)	0.71–2.63 (1.79)	8(1)	0.54
	2-Undecanone	112-12-9	12(12)	0.16–2.01 (0.48)	6(1)	0.09–0.09 (0.09)	5(3)	0.12–0.20 (0.14)	4(1)	0.09
	2-Tridecanone	593-08-8	12(12)	2.49–54.53 (11.00)	3(0)	–	4(2)	1.25–1.73 (1.49)	2(0)	–
	*2*-*Pentadecanone*	*2345-28-0*	*12(12)*	*72,700–1,249,400 (242,200)*	*11(11)*	*120–8,800 (4,900)*	*12(12)*	*2,000–16,800 (6,200)*	*12(12)*	*650–7,800 (1,300)*
	*2*-*Heptadecanone*	*2922-51-2*	*12(12)*	*45,800–862,800 (113,000)*	*11(11)*	*420–12,300 (3,000)*	*12(12)*	*500–15,600 (2,400)*	*12(12)*	*400–14,400 (2,000)*

*For uncalibrated species (in italics), ranges, and medians of peak areas are only provided. Compounds are ordered with respect to increasing retention time*.

**Table 4:** Consumption (uptake) and emission (release) of VOCs by HGC-27, CLS-145, and HSEC cells related to the medium only.

	**VOC**	**CAS**	**HGC-27 vs. Medium *p*-value**	**CLS-145 vs. Medium *p*-value**	**HSEC vs. Medium *p*-value**	**Literature data on VOC emission**		
						**Cell line**	**Profile**	**Cell type**
Uptake	Propanal, 2-methyl	78-84-2	4.9 × 10^−4^	9.8 × 10^−4^	4.9 × 10^−4^	HepG2	↓	Liver cancer Mochalski et al., 2013b
						CALU-1	↓	Lung cancer Filipiak et al., 2008
						NCI-H2087	↓	Lung cancer Sponring et al., 2009
						A549	↓	Lung cancer Filipiak et al., 2010
						HUVEC	↓	Endothelial cells Mochalski et al., 2015
						hFB	↓	Human fibroblasts Filipiak et al., 2010
						SGBS	↓	Adipocytes Mochalski et al., 2019
	2-Propenal, 2-methyl-	78-85-3	4.9 × 10^−4^	9.8 × 10^−4^	4.9 × 10^−4^	A549	↓	Lung cancer Filipiak et al., 2010
						HepG2	↓	Liver cancer Mochalski et al., 2013b
						CALU-1	↓	Lung cancer Filipiak et al., 2008
						NCI-H1666	↓	Lung cancer Sponring et al., 2010
						Lu7466, Lu7387	↓	Lung cancer Schallschmidt et al., 2015
						HUVEC	↓	Endothelial cells Mochalski et al., 2015
	Furan, 2-methyl-	534-22-5	n.s.	9.8 × 10^−4^	n.s.	–	–	–
	Butanal, 3-methyl-	590-86-3	4.9 × 10^−4^	9.8 × 10^−4^	4.9 × 10^−4^	NCI-H2087	↓	Lung cancer Sponring et al., 2009
						NCI-H1666	↓	Lung cancer Sponring et al., 2010
						CALU-1	↓	Lung cancer Filipiak et al., 2008
						A549	↓	Lung cancer Filipiak et al., 2010
						A549	↓	Lung cancer Schallschmidt et al., 2015
						HepG2	↓	Liver cancer Mochalski et al., 2013b
						HUVEC	↓	Endothelial cells Mochalski et al., 2015
						hFB, HBEpC	↓	Human fibroblasts Filipiak et al., 2010
	Butanal, 2-methyl-	96-17-3	4.9 × 10^−4^	9.8 × 10^−4^	4.9 × 10^−4^	NCI-H2087	↓	Lung cancer Sponring et al., 2009
						HUVEC	↓	Endothelial cells Mochalski et al., 2015
						hFB	↓	Human fibroblasts Filipiak et al., 2010
	Furan, 2-ethyl-	3208-16-0	4.9 × 10^−4^	9.8 × 10^−4^	4.9 × 10^−4^	SGBS	↑	Preadipocyte Cells Mochalski et al., 2019
	Dimethyl disulphide DMDS	624-92-0	7.8 × 10^−3^	1.6 × 10^−2^	n.s.	SGBS	↓	Preadipocyte Cells Mochalski et al., 2019
	Hexanal	66-25-1	4.9 × 10^−4^	1.9 × 10^−3^	4.9 × 10^−4^	NCI-H1666	↓	Lung cancer Sponring et al., 2010
						HepG2	↓	Liver cancer Mochalski et al., 2013b
						CALU-1	↓	Lung cancer Filipiak et al., 2008
						HBEpC	↓	Human fibroblasts Filipiak et al., 2010
						HUVEC	↓	Endothelial cells Mochalski et al., 2015
						SGBS	↓	Adipocytes Mochalski et al., 2019
	Heptanal	111-71-7	3.4 × 10^−3^	1.9 × 10^−3^	9.8 × 10^−4^	Lu7466, Lu7387	↓	Lung cancer Schallschmidt et al., 2015
	Furan, 2-Pentyl-	3777-69-3	1.6 × 10^−2^	1.9 × 10^−3^	4.9 × 10^−4^	SGBS	↑	Preadipocyte Cells Mochalski et al., 2019
	Benzaldehyde	100-52-7	2.4 × 10^−3^	9.8 × 10^−4^	4.9 × 10^−4^	SW480, SW1116, NCM460	↓	Colon normal and cancer cells Zimmermann et al., 2007
						HeLa	↓	Cervical carcinoma Nozoe et al., 2015
						Scaber, J82, 5637	↑	Bladde cancer Rodrigues et al., 2018
						HepG2	↓	Liver cancer Mochalski et al., 2013b
						HUVEC	↓	Endothelial cells Mochalski et al., 2015
						hFB	↓	Human fibroblasts Filipiak et al., 2010
	Nonanal	124-19-6	3.7 × 10^−2^	5.9 × 10^−3^	7.8 × 10^−3^	HeLa	↓	Cervical carcinoma Nozoe et al., 2015
						A549	↑	Lung cancer Furuhashi 2020
Release	Ethyl acetate	141-78-6	4.9 × 10^−4^	9.8 × 10^−4^	4.9 × 10^−4^	T-47D, MDA-MB-231, MCF-7	↑	Breast cancer Silva et al., 2017
						A549	↑	Lung cancer Schallschmidt et al., 2015
						HUVEC	↑	Endothelial cells Mochalski et al., 2015
						SGBS	↑	Preadipocyte Cells Mochalski et al., 2019
	2-Pentanone	107-87-9	4.9 × 10^−4^	n.s.	4.9 × 10^−4^	MCF-7	↑	Breast cancer Silva et al., 2017
						HepG2	↑	Liver cancer Mochalski et al., 2013b
						hFB, HBEpC	↑	Human fibroblasts, human epithelial primary cells Filipiak et al., 2010
						A549	↑	Lung cancer Filipiak et al., 2010
						SGBS	↑	Preadipocyte Cells Mochalski et al., 2019
	Ethyl propanoate	105-37-3	n.s.	1.9 × 10^−2^	4.9 × 10^−4^	T-47D, MDA-MB-231, MCF-7	↑	Breast cancer Silva et al., 2017
						HUVEC	↑	Endothelial cells Mochalski et al., 2015
	Toluene	108-88-3	2.4 × 10^−3^	n.s.	n.s.	HUVEC	↑	Endothelial cells Mochalski et al., 2015
						A549	↑	Lung cancer Furuhashi 2020
	1-Butanol, 3-methyl-	123-51-3	2.4 × 10^−3^	2.9 × 10^−3^	4.9 × 10^−4^	RGP, Mm	↑	Melanoma Kwak et al., 2013
						SW1116	↑	Colon cancer Zimmermann et al., 2007
						hFB	↑	Human fibroblasts Filipiak et al., 2010
	1-Butanol, 2-methyl-	137-32-6	1.9 × 10^−3^	n.s.	4.9 × 10^−4^	VGP, Mm	↑	Melanoma Kwak et al., 2013
	Ethyl 2-methylbutyrate	7452-79-1	4.9 × 10^−4^	9.8 × 10^−4^	4.9 × 10^−4^	–	–	–
	2-Heptanone	105-42-0	4.9 × 10^−4^	9.8 × 10^−4^	1.9 × 10^−2^	T-47D, MDA-MB-231, MCF-7	↑	Breast cancer Silva et al., 2017
						HepG2	↑	Liver cancer Mochalski et al., 2013b
	2-Methyl-5-methyl thio furan	2371-70-2	4.9 × 10^−4^	9.8 × 10^−4^	4.9 × 10^−4^	SGBS	↑^***^	Preadipocyte Cells Mochalski et al., 2019
	1-Hexanol, 2-ethyl-	104-76-7	n.s.	4.9 × 10^−3^	1.6 × 10^−2^	A549	↑	Lung cancer Furuhashi 2020
						T-47D, MDA-MB-231, MCF-7	↑	Breast cancer Silva et al., 2017
						NCI-H2087	↑	Lung cancer Sponring et al., 2009
						hFB*	↑	Human fibroblasts Filipiak et al., 2010
						HDF	↑	Human dermalfibroblasts Acevedo et al., 2007
	2-Nonanone	821-55-6	4.9 × 10^−4^	1.6 × 10^−2^	n.s.	SW480, SW1116	↑	Colon cancer Zimmermann et al., 2007
						NCM460	↑	Normal cell lines Zimmermann et al., 2007
						Scaber, J82, 5637	↑	Bladder cancer Rodrigues et al., 2018
						NCIH446	↑	Lung cancer Wang et al., 2012
						HepG2	↑	Liver cancer Mochalski et al., 2013b
						HUVEC	↑	Endothelial cells Mochalski et al., 2015
	2-Undecanone	112-12-9	4.9 × 10^−4^	n.s.	n.s.	HeLa	↑	Cervical carcinoma Nozoe et al., 2015
						J82	↑	Bladder cancer Rodrigues et al., 2018
	2-Tridecanone	593-08-8	4.9 × 10^−4^	n.s.	n.s.	HeLa	↑	Cervical carcinoma Nozoe et al., 2015
						A549, SKMES-1	↑	Lung cancer Wang et al., 2012
						A549, HCIH 446, SK-MEM-1		Lung cancer Yu et al., 2009
	2-Pentadecanone	2345-28-0	4.9 × 10^−4^	1.9 × 10^−2^	1.5 × 10^−3^	PC3, 22RV1, DU145, LNCaP	↑	Prostate cancer Lima et al., 2018
						SW480	↑	Colon cancer Zimmermann et al., 2007
						HeLa	↑	Cervical carcinoma Nozoe et al., 2015
						J82	↑	Bladder cancer Rodrigues et al., 2018
						Scaber, 5637	↓	Bladder cancer Rodrigues et al., 2018
						A549, NCIH446, SKMES-1	↑	Lung cancer Wang et al., 2012
	2-Heptadecanone	2922-51-2	4.9 × 10^−4^	n.s.	n.s.	A549, SKMES-1	↑	Lung cancer Wang et al., 2012

*Outcome of a Wilcoxon signed rank test (n.s., not significant)*.

**Figure 1:**
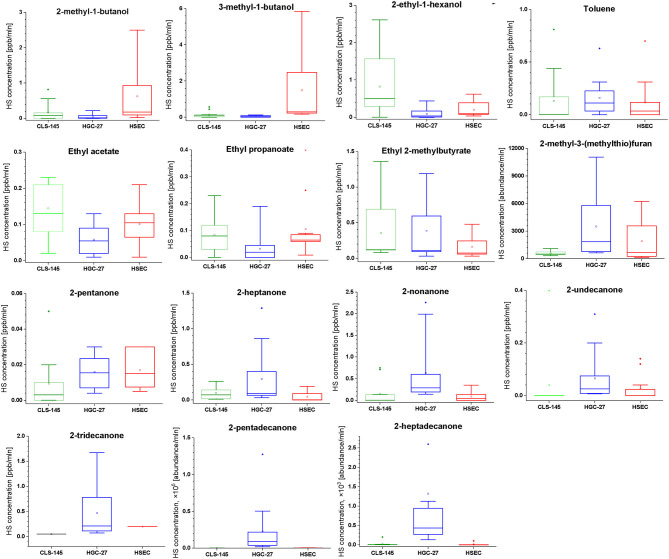
Comparison of the headspace concentrations of VOCs of interest over the cultures of HGC-27, CLS-145, and HSEC cells.

To compare the emissions of VOCs by the cells, the associated signal intensities were normalized to the number of cells in particular cultures. The emission was evaluated using a Wilcoxon signed rank test, the outcome of which is presented in Table 5. The comparison of this emission is presented in Figure 1.

**Table 5:** Comparison of the emission of VOCs by the cells under study.

	**VOC**	**CAS**	**HGC-27 vs. HSEC *p*-value**	**CLS-145 vs. HSEC *p*-value**	**HGC-27 vs. CLS-145 *p*-value**
Release	Ethyl acetate	141-78-6	↓ 2.4 × 10^−3^	↑ 6.9 × 10^−3^	↓ 9.8 × 10^−4^
	2-Pentanone	107-87-9	n.s.	n.s.	n.s.
	Ethyl propanoate	105-37-3	↓ 4.9 × 10^−4^	n.s.	↓ 9.8 × 10^−4^
	Toluene	108-88-3	n.s.	n.s.	n.s.
	1-Butanol, 3-methyl-	123-51-3	↓ 4.9 × 10^−4^	↓ 9.8 × 10^−4^	↓ 2.9 × 10^−3^
	1-Butanol, 2-methyl-	137-32-6	↓ 4.9 × 10^−4^	↓ 1.9 × 10^−3^	↓ 6.9 × 10^−3^
	Ethyl 2-methylbutyrate	7452-79-1	n.s.	↑ 3.2 × 10^−2^	n.s.
	2-Heptanone	105-42-0	↑ 4.9 × 10^−4^	n.s.	n.s.
	*2-Methyl-5-(methyl thio) furan*	*2371*-*70*-*2*	*↑ 4.9* × *10^−4^*	*n.s*.	*↑ 9.8* × *10^−4^*
	1-Hexanol, 2-ethyl-	104-76-7	↓ 9.3 × 10^−3^	↑ 6.9 × 10^−3^	↓ 9.8 × 10^−4^
	2-Nonanone	821-55-6	↑ 4.9 × 10^−4^	n.s.	↑ 9.8 × 10^−4^
	2-Undecanone	112-12-9	↑ 9.8 × 10^−4^	n.s.	↑ 4.2 × 10^−2^
	2-Tridecanone	593-08-8	↑ 4.9 × 10^−4^	n.s.	↑ 9.8 × 10^−4^
	*2-Pentadecanone*	*2345*-*28*-*0*	*↑ 4.9* × *10^−4^*	*n.s*.	*↑ 9.8* × *10^−4^*
	*2-Heptadecanone*	*2922*-*51*-*2*	*↑ 4.9* × *10^−4^*	*n.s*.	*↑ 9.8* × *10^−4^*

*Outcome of a Wilcoxon signed rank test. (n.s., not significant)*.

## Discussion

### Major Findings

Here we present for the first time a comprehensive overview on the volatilomic signature of human HGC-27 and CLS-145 gastric cancer cell lines. Amongst the volatile species detected, 27 showed significant differences compared to the volatiles contained only in the headspace of the cultivating medium, with 12 being produced and 15 being metabolized.

HGC-27 cancer cell lines were found to have significantly altered metabolism in comparison to the normal gastric cells. This was manifested by the increased production of methyl ketones containing an odd number of carbons. Amongst this set of species, three were found exclusively to be produced by this line, namely 2-undecanone, 2-tridecanone, and 2-heptadecanone. Another interesting feature of the HGC-27 volatile footprint is the lowered level of alcohols and esters.

The CLS-145 cells exhibited less pronounced changes in their volatilomic pattern. Their volatile footprint is characterized by the upregulated production of esters and 2-ethyl-hexanol and downregulated production of other alcohols, most probably stemming from the oxidation of aldehydes by ALDHs.

### Volatiles Released and Metabolized by HGC-27, CLS-145, and HSEC Cell Lines

Aldehydes were consumed by all cell lines used in this investigation. The uptake of aldehydes is commonly observed in cultures of human cell lines both normal (Filipiak et al., 2010; Mochalski et al., 2015) and cancerous (Filipiak et al., 2008; Sponring et al., 2009; Mochalski et al., 2013b). Studies reporting production of compounds from this chemical family by human cells are relatively sparse (Furuhashi et al., 2020). The consumption of aldehydes by human cells can be assigned to the expression of aldehyde dehydrogenases (ALDHs) oxidizing aldehydes into their corresponding carboxylic acids (Klyosov, 1996; Crabb et al., 2004). An alternative pathway involves alcohol dehydrogenases (ADHs), which can reversibly reduce aldehydes to alcohols. Indeed, 2-methyl-1-butanol, 3-methyl-1-butanol products resulting from the reduction of 2-methylbutanal, and 3-methylbutanal have been found to be released into the headspace of all cell lines. Moreover, 1-propanol and 2-methyl-1-propanol have also been identified in the headspace of the culture flasks; however, no statistically significant difference in the concentrations of these species was found between media and cell cultures. Perhaps, the production of these volatiles was too low for a sound comparison of their headspace levels given their relatively high levels in the applied medium.

Three heterocyclic compounds metabolized by the cells of interest: 2-methyl-furan, 2-ethyl-furan, and 2-pentyl-furan. However, 2-methyl furan was found to be metabolized only by CLS-145. Two possible pathways of the 2-methylfuran uptake can be proposed. 2-methylfuran may become (i) irreversibly associated with microsomal proteins and/or DNA, or (ii) oxidized to 4-oxo-2-pentenal by cytochrome P450 2E1 (Peterson, 2013). Perhaps the remaining furans undergo the analogous reactions. Furan-containing compounds are rarely reported to be associated with human cells' volatilome. One example was reported to be produced by Simpson-Golabi-Behmel syndrome (SGBS) adipocytes (Mochalski et al., 2019).

Amongst the liberated compounds, ketones are the most numerous, with seven representatives. Intriguingly, all of these are methyl ketones containing an odd number of carbons. Two potential pathways could be responsible for their production: (i) oxidation of secondary alcohols catalyzed by ADHs and/or cytochrome p450 CYP2E1, and (ii) β-oxidation of fatty acids. Although ADHs are the enzymes mostly responsible for ethanol metabolism; they also oxidize secondary, long-chain and cyclic alcohols (Ditlow et al., 1984; Crabb et al., 2004). Consequently, 2-pentanone could stem from 2-pentanol, and 2-heptanone from 2-heptanol. However, none of the potential alcohol substrates for this route was detected in the medium headspace. Nevertheless, they could be produced from respective alkanes via the hydroxylation catalyzed by cytochrome p450, as demonstrated for 2-nonanol (Edwards et al., 2005). As an alternative metabolic route, β-oxidation of fatty acids was also indicated as a source of ketones in humans. For example, 2-ethylhexanoic acid is metabolized to 2-heptanone and 4-heptanone (Walker and Mills, 2001); whereas, 2-pentanone is hypothesized to be formed via β-oxidation of hexanoic acid in the peroxisomal pathway (Walker and Mills, 2014). Moreover, methyl ketones can also be produced spontaneously via the decarboxylation of long chain β-keto acids (Yan et al., 2020). All ketones of interest were reported to be produced by numerous human cancer and normal cell lines. For instance, 2-pentanone was found to be released by human breast cancer line MCF-7(Silva et al., 2017), liver cancer cell line HepG2 (Mochalski et al., 2013b), lung cancer cell line A549 (Filipiak et al., 2010), adipocyte cells SGBS (Mochalski et al., 2019) and human fibroblasts (hFB, HBEpC) (Filipiak et al., 2010). The production of 2-heptanone was observed in liver cancer (HepG2) and lung breast cell cultures (T-47D, MDA-MB-231, MCF-7) (Mochalski et al., 2013b; Silva et al., 2017). Furthermore, 2-nonanone was found to be liberated by cell cultures of colon cancer (SW480, SW1116) (Zimmermann et al., 2007), bladder cancer (J82, 5637) (Rodrigues et al., 2018), liver cancer (HepG2) (Mochalski et al., 2013b), and lung cancer (NCIH446) (Wang et al., 2012). Finally, 2-undecanone, 2-tridecanone, 2-pentadecanone and 2-heptadecanone were found in the cultures of lung cancer (Yu et al., 2009; Wang et al., 2012), bladder cancer (Rodrigues et al., 2018), colon cancer (Zimmermann et al., 2007) and prostate cancer (Lima et al., 2018). It should be stressed here that the production of methyl ketones clearly distinguished HGC-27 cells volatilomic pattern from the other cells investigated. We find that HGC-27 cells released several unique species from this family, including 2-undecanone, 2-tridecanone, and 2-heptadecanone.

Three alcohols were found to be emitted by the cells under investigation: 2-methyl-1-butanol, 3-methyl-1-butanol, and 2-ethyl-1-hexanol, with the exception that the latter was not found to be produced by the HGC-27 cell line. Both 2-methyl-1-butanol and 3-methyl-1-butanol have been reported to be liberated by several cancerous human cell lines (Zimmermann et al., 2007; Kwak et al., 2013) and normal healthy ones (Filipiak et al., 2010). As mentioned above the most probable origin of 2-methyl-1-butanol and 3-methyl-1-butanol is associated with the reduction of 2-methylbutanal and 3-methylbutanal performed by ADHs. An optional pathway employs the hydroxylation of hydrocarbons catalyzed by cytochrome p450 isoforms, e.g., 1A2, 2B6, and 2E1 (Frommer et al., 1970; Edwards et al., 2005; Ortiz de Montellano, 2010). However, the p450 hydroxylation of 2-methyl-butane—a potential substrate for both alcohols under scrutiny—does not occur at primary C-H bonds (Frommer et al., 1970; Ortiz de Montellano, 2010). Consequently, the production of 2-methyl-1-butanol and 3-methyl-1-butanol from this species is unlikely. The release of 2-ethyl-1-hexanol can be attributed to the metabolism of di(2-ethylhexyl) phthalate (DEHP)—a plasticizer used in polyvinyl chloride products (Wahl et al., 2004). In humans, DEHP is rapidly hydrolyzed to mono(2-ethylhexyl) phthalate (MEHP) and 2-ethylhexanol (by cholesterol esterase (CEase), and/or carboxylesterase Ces1e). The latter is then oxidized to 2-ethylhexanoic acid and finally to 2-heptanone and 4-heptanone (Walker and Mills, 2001; Wahl et al., 2004; Saito et al., 2010; Ozaki et al., 2017). Although 4-heptanone was not detected in the headspace of the cell cultures, all investigated cell lines emitted 2-heptanone. 2-ethylhexanol could also be the product of the oxidation of 2-ethyl-hexanal catalyzed by ADHs. Regarding other human cell lines, 2-ethylhexanol was shown to be liberated by A549 lung cancer cell line (Furuhashi et al., 2020).

Three ethyl esters (ethyl acetate, ethyl propanoate, and ethyl 2-methylbutyrate) were found to be produced by all three cell types under investigation. A possible metabolic route leading to the formation of these species involves the esterification reaction employing ethanol and respective carboxylic acids (acetic acid, propanoic acid, and 2-methylbutanoic acid). Acetic acid is a common metabolite found in the human organism, originating from normal human biochemical pathways, such as the Krebs cycle, or by pyruvate metabolism. It could be also produced from ethanol present in the applied medium by a tandem of ADHs and ALDHs. Propanoic acid and 2-methylbutanoic acid were presumably the products of the aforementioned oxidation of propanal and 2-methylbutanal by ALDHs. Although this reaction in the absence of a catalyst is very slow and the products relatively unstable, the aforementioned esters could be formed in the cell cultures and released into the headspace. If so, the production of esters would reflect the activity of ALDHs. Esters of interest are quite common members of human cells volatilome. Ethyl acetate was found to be liberated by human breast and lung cancer cells (Schallschmidt et al., 2015; Silva et al., 2017) as well as endothelial cells and adipocytes (Mochalski et al., 2015, 2019). Ethyl propanoate in turn was detected in the cultures of breast cancer and endothelial cells (Mochalski et al., 2015; Silva et al., 2017).

HGC-27 cells were found to release toluene, the origin of which is unclear. The presence of toluene in human organism is commonly attributed to environmental exposure, or to smoking, but toluene was shown to be liberated by several human cell lines such as A549 lung cancer cells (Furuhashi et al., 2020) and human endothelial cells (HUVEC) (Mochalski et al., 2015).

All cell lines emitted 2-methyl-5-(methyl-thio)-furan. The metabolic route leading to the production of this species is unknown. However, it has been also shown to be released by Simpson-Golabi-Behmel syndrome (SGBS) adipocytes (Mochalski et al., 2019) and liver cancer cells (HepG2) (Mochalski et al., 2013b).

**Figure 2:**
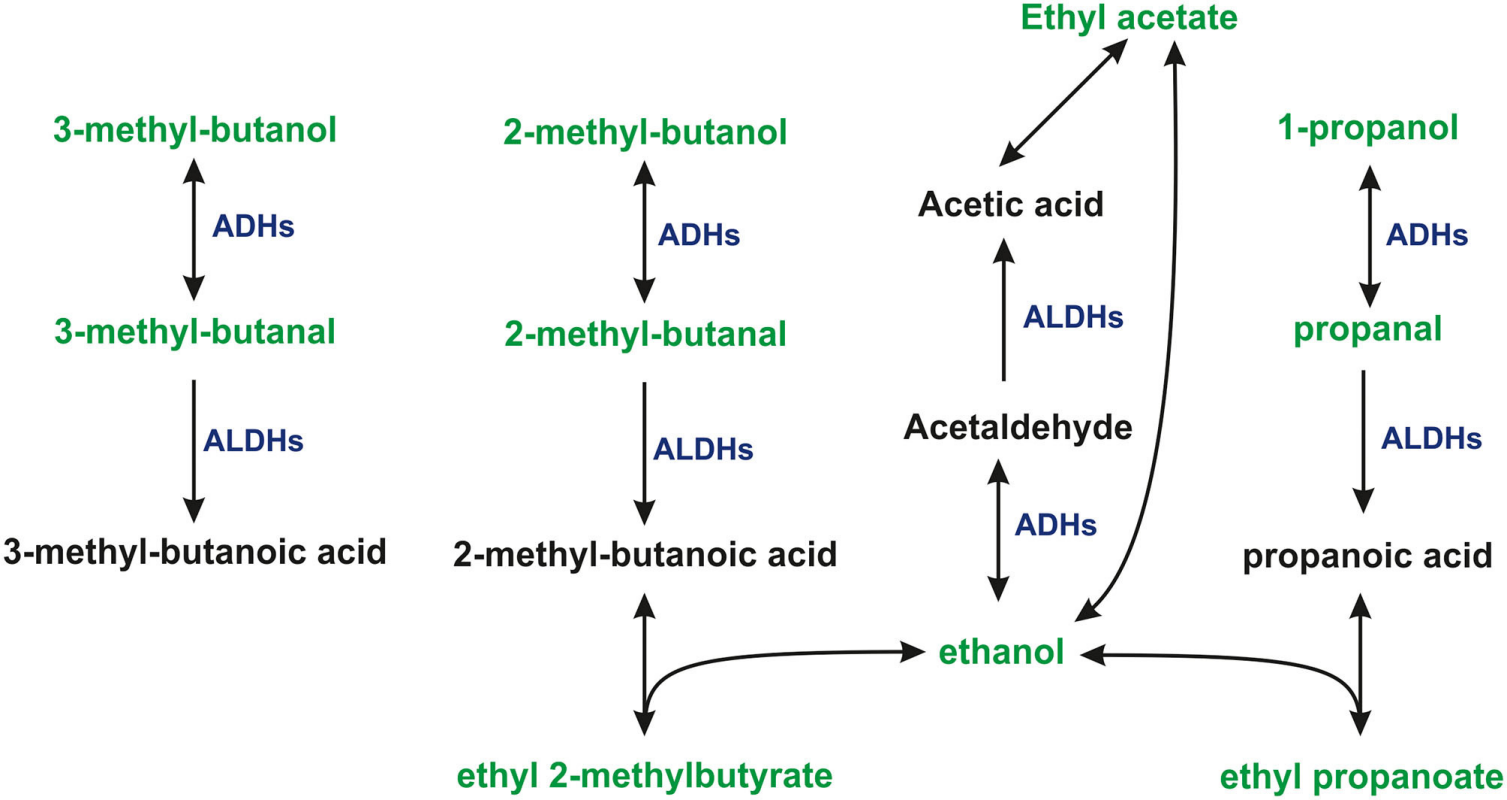
The proposed pathways leading to the consumption, or production of some VOCs under study. 1-propanol and ethanol were present in the headspace of cultures and medium at levels exceeding the dynamic range of the method.

The proposed pathways leading to the consumption or production of some VOCs of interests are presented in Figure 2.

Owing to the inconsistency of the literature data on the potential volatile markers for gastric cancer, it is difficult to relate the results obtained in this study to the results provided by other investigators. It should be stressed here, that this inconsistency is manifested by the fact that each study provides a completely different set of potential biomarkers. The underlying cause of these discrepancies is unknown. Perhaps, issues relating to the biomarkers identification, or the small populations of the enrolled patients/controls could play a significant role. Moreover, the majority of the volatiles we have discovered have not been reported in the literature to be associated with gastric cancer. Only 2-pentanone was found to be emitted in higher amounts from gastric cancer tissue than from normal gastric tissue (Mochalski et al., 2018), which is consistent with this study. Our results provide additional evidence that aldehydes are commonly consumed by human cells in general and by gastric cancer cells in particular. This observation contradicts the results obtained by Kumar et al. (2015), who observed an increase in the levels of some aldehydes including hexanal, heptanal, and nonanal in exhaled air. Perhaps the increase of the aldehydes levels in breath reported by Kumar et al. reflects the body response toward cancer rather than cancer metabolism. Nevertheless, the consumption of aldehydes by cell lines under study questions the usefulness of compounds from this chemical class as potential markers of gastric cancer detected in either breath, urine, or saliva. The volatilomic studies on gastric cancer cells are relatively sparse. To the best of our knowledge, the only line that has been investigated to date is MGC-803 (Zhang et al., 2014). However, none of the species reported to be produced by the MGC-803 cells was found to be emitted by lines investigated in this study. Perhaps different mutations activate or suppress different metabolic pathways in gastric cancer lines. This issue remains to be clarified in future studies.

### Comparison of Chemical Patterns of HGC-27, CLS-145, and HSEC Cell Lines

Within the limits of this approach, some valuable information is extracted concerning the emission of volatiles by the cells of interest. First, the emission of alcohols was significantly lower in cancer cells in comparison to the normal ones. This was particularly pronounced for HGC-27 cells. This observation may be explained by the overexpression of ALDHs. Indeed, aldehyde dehydrogenases have been demonstrated to be significantly elevated in gastric cancer cells (Nishikawa et al., 2013; Wu et al., 2016). Interestingly, the CLS-145 line exhibited a lowered production of 2-methyl-1-butanol and 3-methyl-1-butanol and an increased emission of 2-ethyl-1-hexanol. This finding clearly indicates a different metabolic origin for 2-ethyl-1-hexanol. It is possible that the upregulated production of 2-ethyl-1-hexanol in CLS-145 cells reflects activation of cholesterol esterase (CEase), and/or carboxylesterase (Ces1e) involved in the metabolism of DEHP. Secondly, the most pronounced feature of the HGC-27 volatile footprint is the emission of methyl ketones. Three ketones; 2-undecanone, 2-tridecanone, and 2-heptadecanone were found to be produced exclusively by this cell line. Moreover, the emissions of 2-heptanone, 2-nonanone, and 2-pentadecanone were significantly higher from this line than from the HSEC and CLS-145 lines. Of the ketones, only 2-pentanone was released at similar levels by all cells under investigation. This upregulation of ketones production clearly differentiates HGC-27 from the other cells. One possible explanation of this feature may be the overexpression of ADHs, which convert primary alcohols into aldehydes and secondary alcohols into ketones. Indeed, the total ADH activity has been demonstrated to be significantly elevated in cancer tissues in general (Jelski and Szmitkowski, 2008) and in gastric cancer tissue in particular (Jelski et al., 2008). However, the CLS-145 cells do not exhibit this feature. Alternatively, ketones emission could be triggered by the upregulation of the β-oxidation of fatty acids. Nevertheless, it is not clear which mechanism may be responsible for the overproduction of ketones by the HGC-27 line.

When it comes to esters, the cancer cell lines showed interesting differences. The HGC-27 cell line exhibited lower headspace concentrations of ethyl acetate and ethyl propanoate than found for both HSEC and CLS-145 cell lines; whereas, CLS-145 cells produced significantly higher amounts of ethyl acetate and ethyl 2-methylbutyrate in comparison to HSEC. Perhaps this difference stems from the changes of the ALDHs expression in CLS-145 and HGC-27 lines induced by cancer. If so, CLS-145 cells would be characterized by the upregulated activity of ALDHs. Finally, the HGC-27 line was found to emit greater amounts of 2-methyl-5-(methyl thio)-furan than the other cells.

## Conclusions

Although it is not clear what mechanisms underlie the mentioned changes in the volatilomic profile, our results provide evidence that cancer-related changes in the cells metabolism can be detected via analysis of their chemical volatile signature. Thus, our work has demonstrated that VOCs analysis can be used, as a noninvasive tool for monitoring metabolic changes induced by cancer in the body. This noninvasive approach is not necessarily limited to breath analysis, as it can also easily be adapted to other types of samples, e.g., urine. Moreover, as the volatilomic profile reflects the situation in the circulation, every relevant compound/metabolite produced by the gastric tissue should be detected in breath.

Whether one VOC or a certain set of VOCs found within this study might have value to diagnose gastric cancer via breath and/or urine analyses remains to be clarified in future studies.

Taken together, we have demonstrated that it is possible to differentiate between cancerous and healthy gastric cells using biochemical volatile signatures and that VOC analysis can in principle be used as a noninvasive tool for monitoring metabolic changes in the body, e.g., induced by cancer.

A particular strength of our investigations is the experimental protocol, comprising of measurements in triplicate for each of the four independent experiments, performed by different staff. A further strength is the fact that external confounders including food, medical treatment, smoking, microbiotic colonization and volatile compounds diffusing from plastic ware could be precluded by the glassware-based *in vitro* setting. A limitation in our study design should be raised, namely the general restriction of using cell lines instead of biopsied material for detecting biomarker compounds. However, the use of cell lines allows for more standardized cultivation and, more importantly, the use of two different cell lines minimizes confounding influences by cell line specific effects.

## Data Availability Statement

The raw data supporting the conclusions of this article will be made available by the authors, without undue reservation.

## Author Contributions

PM and AL were the coordinating principal investigators, who designed the study, developed methodology, supervised experiments, and wrote the draft of the manuscript. DŚ performed GC-MS analyses, calibration and validation measurements, and contributed to data processing. AL, AM, and CH performed the cell culture experiments. LM and IK participated in data analysis and interpretation and revised the manuscript. CAM, ML, and HD contributed to the conception, supervision of the study, and manuscript revision. All authors read and approved the final manuscript.

## Conflict of Interest

The authors declare that the research was conducted in the absence of any commercial or financial relationships that could be construed as a potential conflict of interest.

## References

[B1] Acevedo C. A. Sanchez E. Y. Reyes J. G. Young M. E. (2007). Volatile organic compounds produced by human skin cells. Biol. Res. 40, 347–355. 10.4067/S0716-97602007000400009 18449462

[B2] Akagi T. Kimoto T. (1976). Human cell line (Hgc-27) derived from metastatic lymph-node of gastric cancer. Acta Med. Okayama 30, 215–219. 136873

[B3] Amal H. Leja M. Funka K. Skapars R. Sivins A. Ancans G. L.. (2015). Detection of precancerous gastric lesions and gastric cancer through exhaled breath. Gut 65, 400–4007. 10.1136/gutjnl-2014-308536 25869737

[B4] Amann A. Costello B. Miekisch W. Schubert J. Buszewski B. Pleil J.. (2014). The human volatilome: volatile organic compounds (VOCs) in exhaled breath, skin emanations, urine, feces and saliva. J. Breath Res. 8, 034001. 10.1088/1752-7155/8/3/034001 24946087

[B5] Antweiler R. C. (2015). Evaluation of statistical treatments of left-censored environmental data using coincident uncensored data sets. II. Group Comparisons. Environ. Sci. Technol. 49, 13439–13446. 10.1021/acs.est.5b02385 26490190

[B6] Beauchamp J. Davies C. Pleil J. E. (2020). Breathborne Biomarkers and the Human Volatilome. Amsterdam: Elsevier.

[B7] Broza Y. Y. Mochalski P. Ruzsanyi V. Amann A. Haick H. (2015). Hybrid volatolomics and disease detection. Angew. Chem. Int. Ed. 54, 11036–11048. 10.1002/anie.201500153 26235374

[B8] Buszewski B. Ulanowska A. Ligor T. Jackowski M. Klodzinska E. Szeliga J. (2008). Identification of volatile organic compounds secreted from cancer tissues and bacterial cultures. J. Chromatogr. B Anal. Technol. Biomed. Life Sci. 868, 88–94. 10.1016/j.jchromb.2008.04.038 18490205

[B9] Crabb D. W. Matsumoto M. Chang D. You M. (2004). Overview of the role of alcohol dehydrogenase and aldehyde dehydrogenase and their variants in the genesis of alcohol-related pathology. Proc. Nutr. Soc. 63, 49–63. 10.1079/PNS2003327 15099407

[B10] del Rio R. F. O'Hara M. E. Holt A. Pemberton P. Shah T. Whitehouse T.. (2015). Volatile biomarkers in breath associated with liver cirrhosis - comparisons of pre- and post-liver transplant breath samples. Ebiomedicine 2, 1243–1250. 10.1016/j.ebiom.2015.07.027 26501124 PMC4588000

[B11] Ditlow C. C. Holmquist B. Morelock M. M. Vallee B. L. (1984). Physical and enzymatic properties of a class II alcohol dehydrogenase isozyme of human liver: pi-ADH. Biochemistry 23, 6363–6368. 10.1021/bi00321a012 6397223

[B12] Durán-Acevedo C. M. Jaimes-Mogollón A. L. Gualdrón-Guerrero O. E. Welearegay T. G. Martinez-Marín J. D. Caceres-Tarazona J. M.. (2018). Exhaled breath analysis for gastric cancer diagnosis in Colombian patients. Oncotarget 9, 28805–28817. 10.18632/oncotarget.25331 29988892 PMC6034740

[B13] Edwards J. E. Rose R. L. Hodgson E. (2005). The metabolism of nonane, a JP-8 jet fuel component, by human liver microsomes, P450 isoforms and alcohol dehydrogenase and inhibition of human P450 isoforms by JP-8. Chem. Biol. Interact. 151, 203–211. 10.1016/j.cbi.2004.12.003 15733541

[B14] Filipiak W. Sponring A. Filipiak A. Ager C. Schubert J. Miekisch W.. (2010). TD-GC-MS analysis of volatile metabolites of human lung cancer and normal cells *in vitro*. Cancer Epidemiol. Biomarkers Prev. 19, 182–195. 10.1158/1055-9965.EPI-09-0162 20056637

[B15] Filipiak W. Sponring A. Mikoviny T. Ager C. Schubert J. Miekisch W.. (2008). Release of volatile organic compounds (VOCs) from the lung cancer cell line CALU-1 *in vitro*. Cancer Cell Int. 8, 17. 10.1186/1475-2867-8-17 19025629 PMC2639533

[B16] Frommer U. Ullrich V. Stauding H. (1970). Hydroxylation of Aliphatic Compounds by Liver Microsomes 1. Distribution Pattern of Isomeric Alcohols. Hoppe Seylers Z Physiol Chem. 351, 903–12. 10.1515/bchm2.1970.351.2.903 4393760

[B17] Furuhashi T. Ishii R. Onishi H. Ota S. (2020). Elucidation of biochemical pathways underlying VOCs production in A549 cells. Front. Mol. Biosci. 7, 116. 10.3389/fmolb.2020.00116 32695794 PMC7338772

[B18] Haick H. Broza Y. Y. Mochalski P. Ruzsanyi V. Amann A. (2014). Assessment, origin, and implementation of breath volatile cancer markers. Chem. Soc. Rev. 43, 1423–1449. 10.1039/C3CS60329F 24305596 PMC4909138

[B19] Huber W. (2003). Basic calculations about the limit of detection and its optimal determination. Accred. Qual. Assur. 8, 213–217. 10.1007/s00769-003-0626-8

[B20] Jelski W. Chrostek L. Zalewski B. Szmitkowski M. (2008). Alcohol dehydrogenase (ADH) isoenzymes and aldehyde dehydrogenase (ALDH) activity in the sera of patients with gastric cancer. Dig. Dis. Sci. 53, 2101–2105. 10.1007/s10620-007-0135-4 18231859

[B21] Jelski W. Szmitkowski M. (2008). Alcohol dehydrogenase (ADH) and aldehyde dehydrogenase (ALDH) in the cancer diseases. Clin. Chim. Acta 395, 1–5. 10.1016/j.cca.2008.05.001 18505683

[B22] Jmour O. Belaid A. Mghirbi F. Behi K. Doghri R. Benna F. (2017). Gastric metastasis of bilateral breast cancer. J. Gastrointest. Oncol. 8, E16–E20. 10.21037/jgo.2016.10.03 28280631 PMC5334049

[B23] Klyosov A. A. (1996). Kinetics and specificity of human liver aldehyde dehydrogenases toward aliphatic, aromatic, and fused polycyclic aldehydes. Biochemistry 35, 4457–4467. 10.1021/bi9521102 8605195

[B24] Kumar S. Huang J. Z. Abbassi-Ghadi N. Mackenzie H. A. Veselkov K. A. Hoare J. M.. (2015). Mass spectrometric analysis of exhaled breath for the identification of volatile organic compound biomarkers in esophageal and gastric adenocarcinoma. Ann. Surg. 262, 981–990. 10.1097/SLA.0000000000001101 25575255

[B25] Kumar S. Huang J. Z. Cushnir J. R. Spanel P. Smith D. Hanna G. B. (2012). Selected ion flow tube-ms analysis of headspace vapor from gastric content for the diagnosis of gastro-esophageal cancer. Anal. Chem. 84, 9550–9557. 10.1021/ac302409a 23035898

[B26] Kwak J. Gallagher M. Ozdener M. H. Wysocki C. J. Goldsmith B. R. Isamah A.. (2013). Volatile biomarkers from human melanoma cells. J. Chromatogr. B Anal. Technol. Biomed. Life Sci. 931, 90–96. 10.1016/j.jchromb.2013.05.007 23770738

[B27] Lima A. R. Araujo A. M. Pinto J. Jeronimo C. Henrique R. Bastos M. D.. (2018). Discrimination between the human prostate normal and cancer cell exometabolome by GC-MS. Sci Rep. 8, 5539. 10.1038/s41598-018-23847-9 29615722 PMC5882858

[B28] Mochalski P. Agapiou A. Statheropoulos M. Amann A. (2012). Permeation profiles of potential urine-borne biomarkers of human presence over brick and concrete. Analyst 137, 3278–3285. 10.1039/c2an35214a 22662321

[B29] Mochalski P. Al-Zoairy R. Niederwanger A. Unterkofler K. Amann A. (2014). Quantitative analysis of volatile organic compounds released and consumed by rat L6 skeletal muscle cells *in vitro*. J. Breath Res. 8, 046003. 10.1088/1752-7155/8/4/046003 25307263 PMC4913865

[B30] Mochalski P. Diem E. Unterkofler K. Mundlein A. Drexel H. Mayhew C. A.. (2019). *In vitro* profiling of volatile organic compounds released by Simpson-Golabi-Behmel syndrome adipocytes. J. Chromatogr. B Anal. Technol. Biomed. Life Sci. 1104, 256–261. 10.1016/j.jchromb.2018.11.028 30537625

[B31] Mochalski P. King J. Klieber M. Unterkofler K. Hinterhuber H. Baumann M.. (2013a). Blood and breath levels of selected volatile organic compounds in healthy volunteers. Analyst 138, 2134–2145. 10.1039/c3an36756h 23435188 PMC4909136

[B32] Mochalski P. Leja M. Gasenko E. Skapars R. Santare D. Sivins A.. (2018). *Ex vivo* emission of volatile organic compounds from gastric cancer and non-cancerous tissue. J. Breath Res. 12, 046005. 10.1088/1752-7163/aacbfb 29893713

[B33] Mochalski P. Sponring A. King J. Unterkofler K. Troppmair J. Amann A. (2013b). Release and uptake of volatile organic compounds by human hepatocellular carcinoma cells (HepG2) *in vitro*. Cancer Cell Int. 13, 72. 10.1186/1475-2867-13-72 23870484 PMC3717104

[B34] Mochalski P. Theurl M. Sponring A. Unterkofler K. Kirchmair R. Amann A. (2015). Analysis of volatile organic compounds liberated and metabolised by human umbilical vein endothelial cells (HUVEC) *in vitro*. Cell Biochem. Biophys. 71, 323–329. 10.1007/s12013-014-0201-4 25123840 PMC4289529

[B35] Nakhleh M. K. Amal H. Jeries R. Broza Y. Y. Aboud M. Gharra A.. (2017). Diagnosis and classification of 17 diseases from 1404 subjects via pattern analysis of exhaled molecules. ACS Nano 11, 112–125. 10.1021/acsnano.6b04930 28000444 PMC5269643

[B36] Nishikawa S. Konno M. Hamabe A. Hasegawa S. Kano Y. Ohta K.. (2013). Aldehyde dehydrogenase(high) gastric cancer stem cells are resistant to chemotherapy. Int. J. Oncol. 42, 1437–1442. 10.3892/ijo.2013.1837 23440340

[B37] Nozoe T. Goda S. Selyanchyn R. Wang T. Nakazawa K. Hirano T.. (2015). *In vitro* detection of small molecule metabolites excreted from cancer cells using a Tenax TA thin-film microextraction device. J. Chromatogr. B Anal. Technol. Biomed. Life Sci. 991, 99–107. 10.1016/j.jchromb.2015.04.016 25932789

[B38] Ortiz de Montellano P. R. (2010). Hydrocarbon hydroxylation by cytochrome P450 enzymes. Chem. Rev. 110, 932–948. 10.1021/cr9002193 19769330 PMC2820140

[B39] Ozaki H. Sugihara K. Watanabe Y. Moriguchi K. Uramaru N. Sone T.. (2017). Comparative study of hydrolytic metabolism of dimethyl phthalate, dibutyl phthalate and di(2-ethylhexyl) phthalate by microsomes of various rat tissues. Food Chem. Toxicol. 100, 217–224. 10.1016/j.fct.2016.12.019 28007454

[B40] Peterson L. A. (2013). Reactive metabolites in the biotransformation of molecules containing a furan ring. Chem. Res. Toxicol. 26, 6–25. 10.1021/tx3003824 23061605 PMC3574180

[B41] Rodrigues D. Pinto J. Araujo A. M. Monteiro-Reis S. Jeronimo C. Henrique R.. (2018). Volatile metabolomic signature of bladder cancer cell lines based on gas chromatography-mass spectrometry. Metabolomics 14, 62. 10.1007/s11306-018-1361-9 30830384

[B42] Saito T. Hong P. Tanabe R. Nagai K. Kato K. (2010). Enzymatic hydrolysis of structurally diverse phthalic acid esters by porcine and bovine pancreatic cholesterol esterases. Chemosphere 81, 1544–1548. 10.1016/j.chemosphere.2010.08.020 20822795

[B43] Schallschmidt K. Becker R. Jung C. Rolff J. Fichtner I. Nehls I. (2015). Investigation of cell culture volatilomes using solid phase micro extraction: options and pitfalls exemplified with adenocarcinoma cell lines. J. Chromatogr. B Analyt. Technol. Biomed. Life Sci. 1006, 158–166. 10.1016/j.jchromb.2015.10.004 26551208

[B44] Silva C. L. Perestrelo R. Silva P. Tomas H. Camara J. S. (2017). Volatile metabolomic signature of human breast cancer cell lines. Sci. Rep. 7, 43969. 10.1038/srep43969 28256598 PMC5335623

[B45] Sponring A. Filipiak W. Ager C. Schubert J. Miekisch W. Amann A.. (2010). Analysis of volatile organic compounds (VOCs) in the headspace of NCI-H1666 lung cancer cells. Cancer Biomark. 7, 153–161. 10.3233/CBM-2010-0182 21263191 PMC12922879

[B46] Sponring A. Filipiak W. Mikoviny T. Ager C. Schubert J. Miekisch W.. (2009). Release of volatile organic compounds from the lung cancer cell line NCI-H2087 *in vitro*. Anticancer Res. 29, 419–426. 19331181

[B47] Wahl H. G. Hong Q. Hildenbrand S. Risler T. Luft D. Liebich H. (2004). 4-Heptanone is a metabolite of the plasticizer di(2-ethylhexyl) phthalate (DEHP) in haemodialysis patients. Nephrol. Dial. Transplant. 19, 2576–2583. 10.1093/ndt/gfh425 15280519

[B48] Walker V. Mills G. A. (2001). Urine 4-heptanone: a beta-oxidation product of 2-ethylhexanoic acid from plasticisers. Clin. Chim. Acta 306, 51–61. 10.1016/S0009-8981(01)00390-4 11282094

[B49] Walker V. Mills G. A. (2014). 2-pentanone production from hexanoic acid by penicillium roqueforti from blue cheese: is this the pathway used in humans? Sci. World J. 2014, 215783. 10.1155/2014/215783 25143966 PMC3985342

[B50] Wang Y. Hu Y. Wang D. Yu K. Wang L. Zou Y.. (2012). The analysis of volatile organic compounds biomarkers for lung cancer in exhaled breath, tissues and cell lines. Cancer Biomark. 11, 129–137. 10.3233/CBM-2012-00270 23144150 PMC13016210

[B51] Wu D. Mou Y. P. Chen K. Cai J. Q. Zhou Y. C. Pan Y.. (2016). Aldehyde dehydrogenase 3A1 is robustly upregulated in gastric cancer stem-like cells and associated with tumorigenesis. Int. J. Oncol. 49, 611–622. 10.3892/ijo.2016.3551 27279633

[B52] Xu Z. Q. Broza Y. Y. Ionsecu R. Tisch U. Ding L. Liu H.. (2013). A nanomaterial-based breath test for distinguishing gastric cancer from benign gastric conditions. Br. J. Cancer 108, 941–950. 10.1038/bjc.2013.44 23462808 PMC3590679

[B53] Yan Q. Simmons T. R. Cordell W. T. Lozada N. J. H. Breckner C. J. Chen X. (2020). Metabolic engineering of β-oxidation to leverage thioesterases for production of 2-heptanone, 2-nonanone and 2-undecanone. Metab. Eng. 61, 335–343. 10.1016/j.ymben.2020.05.008 32479802 PMC7501266

[B54] Yu J. Wang D. Wang L. Wang P. Hu Y. J. Ying K. J. (2009). Detection of lung cancer with volatile organic biomarkers in exhaled breath and lung cancer cells. Olfaction and Electronic Nose, Proceedings 1137, 198 10.1063/1.3156506

[B55] Zhang Y. Gao G. Liu H. Fu H. Fan J. Wang K.. (2014). Identification of volatile biomarkers of gastric cancer cells and ultrasensitive electrochemical detection based on sensing interface of Au-Ag alloy coated MWCNTs. Theranostics 4, 154–162. 10.7150/thno.7560 24465273 PMC3900800

[B56] Zimmermann D. Hartmann M. Moyer M. P. Nolte J. Baumbach J. I. (2007). Determination of volatile products of human colon cell line metabolism by GC/MS analysis. Metabolomics 3, 13–17. 10.1007/s11306-006-0038-y

